# Transmission ratio distortion is frequent in *Arabidopsis thaliana* controlled crosses

**DOI:** 10.1038/s41437-018-0107-9

**Published:** 2018-06-28

**Authors:** Danelle K. Seymour, Eunyoung Chae, Burak I. Arioz, Daniel Koenig, Detlef Weigel

**Affiliations:** 1 Department of Molecular Biology, Max Planck Institute for Developmental Biology, 72076 Tübingen, Germany; 2 Present Address: Department of Ecology and Evolutionary Biology, University of California, Irvine, CA USA; 3 Present Address: Department of Botany and Plant Sciences, University of California, Riverside, CA USA

**Keywords:** Plant genetics, Genetic linkage study

## Abstract

The equal probability of transmission of alleles from either parent during sexual reproduction is a central tenet of genetics and evolutionary biology. Yet, there are many cases where this rule is violated. The preferential transmission of alleles or genotypes is termed transmission ratio distortion (TRD). Examples of TRD have been identified in many species, implying that they are universal, but the resolution of species-wide studies of TRD are limited. We have performed a species-wide screen for TRD in over 500 segregating F_2_ populations of *Arabidopsis thaliana* using pooled reduced-representation genome sequencing. TRD was evident in up to a quarter of surveyed populations. Most populations exhibited distortion at only one genomic region, with some regions being repeatedly affected in multiple populations. Our results begin to elucidate the species-level architecture of biased transmission of genetic material in *A. thaliana*, and serve as a springboard for future studies into the biological basis of TRD in this species.

## Introduction

At the genetic level, evolution is the change in the frequency of allelic variants in a population over time, which can be caused by several different evolutionary forces, including selection. While in many cases the strength of selection is too low for these changes to be detected within a few generations, a unique opportunity to directly study such changes is offered in cases where selection coefficients are high. In such a situation, competition between alleles can be seen already in the distribution of heterozygous progeny (a/A). It is manifested as a deviation from the 1:2:1 Mendelian ratio of diploid genotypes (a/a, a/A, A/A), termed transmission ratio distortion (TRD). Deviation from this ratio has important implications for population dynamics. Because TRD arises from the biased segregation of alleles, it has been suggested that TRD may be a major contributor to the formation of reproductive barriers (Frank 1991; Hurst and Pomiankowski 1991; Orr and Irving 2005).

Although the term “transmission ratio distortion” was only coined in 1968 (Dunn and Bennett 1968), examples of TRD were identified as early as 1928 in *Drosophila obscura*, shortly after the rediscovery of Mendel’s laws (Gershenson 1928). Because sexual dimorphism is common, many of the earliest known cases were discovered because the sex ratio deviated greatly from 1:1 (reviewed in Zimmering et al. 1970). These loci were readily identified without molecular biology assays because biased segregation of sex chromosomes perturbed the sex ratio in subsequent generations (Sturtevant and Dobzhansky 1936; Zimmering et al. 1970). Since sex ratio distortion was first observed, work in a number of species has revealed a range of both meiotic and post-meiotic processes that can give rise to TRD. These processes include non-random segregation of gametes during meiosis, post-meiotic gamete dysfunction or differential gamete success, and differential zygotic fitness (reviewed in Cutter 2012; Lindholm et al. 2016; Rieseberg and Blackman 2010). While instances of each have been characterized, it is still unclear whether meiotic or post-meiotic mechanisms predominate.

TRD has been observed both in natural populations and controlled crosses in a wide range of species (McLaughlin and Malik 2017). With the advent of molecular genotyping, reported cases of TRD dramatically increased and non-random segregation of genetic markers is no longer a surprising feature of mapping populations. Examples of meiotic dysfunction (Buckler et al. 1999; Fishman and Saunders 2008; Rhoades 1942), post-meiotic gamete dysfunction (Koide et al. 2008; Kubo et al. 2011, 2016; Long et al. 2008; Moyle et al. 2006), differential gamete success (Diaz and Macnair 1999; Snow et al. 2000), and differential zygotic fitness (Agorio et al. 2017; Alcázar et al. 2009; Bikard et al. 2009; Bomblies et al. 2007; Chae et al. 2014; Durand et al. 2012; Moyle and Nakazato 2009; Plötner et al. 2017; Vlad et al. 2010) have all been characterized in plants. A correlation between the degree of divergence and the probability of observing TRD in a specific cross has been reported, but this relationship seems to vary by species (Jenczewski et al. 1997; Leppala et al. 2013; Matsubara et al. 2011; Moyle and Nakazato 2010; Moyle et al. 2004; Salomé et al. 2012; Zamir and Tadmor 1986).

Surprisingly, there are few cases where the incidence of TRD in a species has been systematically interrogated. Using advanced multi-parent mapping populations, work in *Drosophila melanogaster* and in *Zea mays* has shown that TRD is readily segregating within a species (Corbett-Detig et al. 2013; McMullen et al. 2009). In both species, these advanced populations were developed from a limited number of founding genotypes. The *D. melanogaster* population was developed from eight genetically distinct lines and natural strains were found to carry an average of 1.15 loci with negative epistatic effects on fitness (Corbett-Detig et al. 2013). Similarly, there was evidence for TRD in each segregating family of the maize population, comprising 26 genetically distinct parents (McMullen et al. 2009). A high incidence of genetic incompatibility (24%) was also found to segregate in a panel of *Saccharomyces cerevisiae* crosses derived from 27 parental strains (Hou et al. 2015). Here, progeny were screened for viability in a range of environmental conditions and an association with TRD was demonstrated for a single cross. One limitation to surveying the incidence of TRD in a large collection of segregating populations is that genotyping thousands of individuals can still be costly. Genotyping pools of individuals to estimate allele frequencies can be much more cost effective (reviewed in Schlötterer et al. 2014). This strategy, commonly referred to as Pool-seq, has been utilized to survey deviations in allele frequency in both natural and segregating populations and to map QTL in pools of individuals from controlled crosses (reviewed in Schlotterer et al. 2014).

In *A. thaliana*, segregation distortion due to partially or fully recessively acting alleles has been observed repeatedly in different experimental population designs (Alonso-Blanco et al. 1998; Balasubramanian et al. 2009; Lister and Dean 1993; Loudet et al. 2002; Mitchell-Olds 1995; Salomé et al. 2012; Simon et al. 2008; Törjék et al. 2008; Werner et al. 2005). The largest published study to date in *A. thaliana* examined segregation distortion in 17 F_2_ populations, over half of which exhibited evidence of distortion (Salomé et al. 2012). Because *A. thaliana* is typically a self-fertilizing species (Bomblies et al. 2010), its preference for inbreeding facilitates the detection of intraspecific distortion, since accessions collected from nature are typically homozygous throughout the genome. Cross-fertilization between accessions removes an allele from its native, homozygous context, thus creating an opportunity for biased transmission.

We have surveyed over 500 segregating F_2_ populations for TRD in order to characterize the incidence of biased transmission within a single species. Segregating F_2_ populations were derived from intercrossing 80 distinct, resequenced *A. thaliana* accessions spanning the Eurasian range of the species (Cao et al. 2011). For this large survey, populations were genotyped using a reduced-representation Pool-seq approach to estimate allelic ratios. In addition to documenting the prevalence of TRD in *A. thaliana*, we have also begun to dissect the population-wide genetic architecture of TRD in this species.

## Materials and methods

### Germplasm

The F_2_ populations were generated by intercrossing 80 natural *Arabidopsis thaliana* accessions with whole-genome resequencing information (Cao et al. 2011). Intercrossing was facilitated by induced male sterility which was achieved by artificial miRNA (amiR) mediated knock-down of the floral homeotic gene *APETALA3* (*AP3*) (Chae et al. 2014). One half of F_1_ plants were transgene-free and able to produce F_2_ progeny through self-fertilization, as each original female grandparent was hemizygous for the amiR transgene. In total, 583 F_2_ populations were generated using 67 of the 80 natural accessions as the female grandparent. Each female grandparent carried the amiR-*AP3* transgene to induce male sterility. All 80 accessions were used as the male grandparent, and on average, each grandparent contributed to 14.7 F_2_ populations. Germplasm information can be found in Table S1 and grandparental seed availability is listed in Table S2.

### Growth conditions

At least 300 individuals from each F_2_ population were sown onto 0.5× MS medium (0.7% agar; pH 5.6). Prior to plating, seeds were gas sterilized for 16 h using 40 ml of household bleach (1–4%) and 1.5 ml of concentrated HCl. Seeds were stratified at 4 °C in the dark for 8 days and then plates were shifted to 23 °C long day conditions (16 h light:8 h dark). After 5 days, seedlings were harvested in bulk and flash frozen in liquid nitrogen.

### DNA extraction and GBS library preparation

DNA was extracted from each pool of F_2_ individuals using a CTAB procedure (2% CTAB, 1.4 M NaCl, 100 mM Tris (pH 8), 20 mM EDTA (pH 8)) (Springer 2010). DNA integrity was confirmed by gel electrophoresis, and DNA quantification was performed using the Qubit fluorimeter (Qubit BR assay) (Thermo Fisher Scientific, Waltham, MA). For library preparation, 300 ng of each DNA sample were diluted in 27 μl. Restriction enzyme-mediated reduced-representation libraries were generated using *KpnI*, which is predicted to cleave the *A. thaliana* reference genome into 8366 fragments. The library preparation protocol is detailed in Rowan et al. (2017). Briefly, DNA was digested and then ligated to barcoded adapter sequences with sticky ends complementary to the *KpnI* cleavage site. After ligation, 96 barcoded samples were pooled and then sheared using the Covaris S220 instrument (Covaris, Woburn, MA). Next, end-repair, dA-tailing, a second universal adapter ligation, and PCR enrichment were performed using the Illumina compatible NEBNext DNA Library Prep Master Mix Set (NEB, Ipswich, MA). Library quality was determined using the Agilent 2100 Bioanalyzer (DNA 1000 kit) (Agilent, Santa Clara, CA) and libraries were normalized (10 nM) based on library quantification (ng/μl) and mean fragment length. Sequencing was performed on the Illumina HiSeq 2000 (Illumina, San Diego, CA). Adapter sequences can be found in Rowan et al. (2017).

### SNP identification and allele frequency estimation

SHORE software (v0.9.0) (Ossowski et al. 2008) was used for all analyses described in this section. Sequencing reads were barcode sorted and quality filtered. During quality filtering the restriction enzyme overhang was also trimmed using SHORE import. Reads for each bulked population were then aligned to the TAIR10 reference genome allowing for two mismatches using SHORE mapflowcell. After alignment, SNPs were called with SHORE qVar using default parameters. Read counts for both the reference and non-reference base were extracted for each polymorphic position. SNPs were filtered further using the grandparental whole-genome information and read counts for the female grandparental allele were output only for positions expected to be segregating between the two initial grandparents based on the resequencing data (Cao et al. 2011). The allele frequency of the female grandparental allele was calculated for each polymorphic position as the number of reads containing the female grandparental allele divided by the total number of reads covering that position.

### Modeling of allele frequency and significance testing for allelic distortion

High read coverage was sought for each library to enable accurate allele frequency estimation. The realized median coverage of the population bulks was 78×. The distribution of read coverage per library is shown in Fig. S1A.

Even with high read coverage, allele frequency estimates were still noisy. To generate accurate allele frequency estimates, the allele frequency was modeled in 5 Mb sliding windows (0.5 Mb steps). We used a beta-binomial model to account for variation in the true allele frequency, as well as stochastic variation that arises from read sampling. From the optimized model we extracted the alpha and beta parameters from each genomic window. These parameters describe the shape of the probability distribution in each window, and from these parameters the mean allele frequency, as well as the 95% confidence intervals (CI) were estimated. Using these estimates, a non-parametric statistical test was performed to assess whether the allele frequency estimates were significantly different from 50%, the expected frequency for non-distorted genomic regions. A false discovery correction (FDR) was performed to account for the number of genomic windows tested per population (*n* = 240). After allele frequency estimation, quality control measures culled low-quality bulks. Populations were excluded from subsequent analysis for the following reasons: (1) having a genome-wide average allele frequency greater than 0.75, (2) exhibiting either CI larger than 0.40 or noisy CI across the genome (standard deviation of CI width greater than 0.15), or (3) displaying three or more chromosomes with windows that did not attain model convergence. After quality control, 492 populations remained for subsequent analyses.

### Identification of distorted regions

Two thresholds were used to identify significantly distorted genomic windows. The first approach utilized *p*-value estimates from the non-parametric statistical test performed on each window. False discovery rate (FDR) corrections were applied to account for the number of tested genomic windows (*n* = 240, *p* < 0.05). Distorted populations were required to have at least five adjacent genomic windows on the biased chromosome with significant FDR corrected *p*-values. Populations with statistically significant segregation distortion are listed in Table S1.

The second, less conservative approach identified outliers by calculating *Z*-scores for each genomic window relative to the mean allele frequency of all surveyed F_2_ populations (0.5029). Allele frequencies for each window were derived from the beta-binomial model predictions. Genomic windows with allele frequency estimates greater than 2.5 times the population-wide standard deviation (0.0382) were considered to be distorted. A distorted F_2_ population was required to contain five genomic windows with significant *Z*-scores on the chromosomes containing the locus of interest. Distorted populations identified using extreme *Z*-scores are listed in Table S1.

### Interval identification using whole-genome resequencing

Six F_2_ populations displayed severe distortion at one of six distinct genomic regions (Fig. S2). 1500 individuals were sown from each of these six populations onto 0.5× MS medium (0.7% agar; pH 5.6) as described for the initial screen. DNA was extracted from each population bulk using a standard CTAB preparation (2% CTAB, 1.4 M NaCl, 100 mM Tris (pH 8), 20 mM EDTA (pH 8)). Illumina TruSeq libraries were prepared according to manufacturer’s guidelines using 1 μg of starting material per population. Libraries were sequenced on an Illumina HiSeq 3000 instrument (Illumina, San Diego, CA). Twenty-one nucleotide long k-mers were identified directly from the short reads using jellyfish (v2.2.3) (Marcais and Kingsford 2011) with the following arguments: -m 21 -s 300M -t 10 -C. Not only does jellyfish identify all unique k-mers, but it also calculates the occurrence, or coverage, of each k-mer. The distribution of 21-mer coverages is shown in Figure S3 for each population. Any 21-mer sequence shared between grandparents should occur at the average genome-wide coverage, and when we plotted 21-mer frequencies, we found a major peak of 21-mer coverage around 40×, the average per-population whole-genome coverage, in all six populations, as expected (Fig. S3). In contrast, 21-mers present in only one of the two parents should have approximately half as much coverage, and a second peak, resulting from a much smaller number of 21-mers, was apparent in all populations as well (Fig. S3). 21-mers found in only one of the two grandparental genomes (coverage < 25×) were aligned to the TAIR10 genome using bwa aln (Li and Durbin 2009). Only perfect matches were allowed. A 1 Mb sliding window (50 kb steps) was used to plot the 21-mer coverage across the distorted chromosome in each population. Regions of the genome with reduced coverage of 21-mers are located within the candidate interval (Fig. S2). Interval boundaries were delineated by merging all windows with values within 1× coverage of the minimal window in the candidate region.

### Interval identification for distortion bulked segregant analysis

Bulked segregant analysis (Michelmore et al. 1991) was used to narrow the candidate intervals for Star-8, ICE49, and ICE63. Sequencing reads from the original screen were combined for all distorted populations sharing the grandparent of interest, resulting in a distorted bulk. Those that shared the grandparent, but did not exhibit distortion, were combined separately, resulting in a normal bulk. Positions segregating between the grandparent of interest and all other members of the bulk were identified. The positions segregating in the distorted bulk are not shared with those segregating in the normal bulk. By combining reads from multiple populations, a median of 806 to 1135× coverage was achieved at each segregating position. Candidate intervals were calculated from the maximally distorted position to any flanking segregating site that was within 5% of the peak allele frequency (Table S3).

## Results

### Frequent segregation distortion in intraspecific *A. thaliana* F_2_ populations

The incidence of TRD was surveyed in 583 F_2_ populations generated from naturally inbred accessions that represent much of the Eurasian genetic diversity in *A. thaliana* (Cao et al. 2011). The studied F_2_ populations were derived from crosses between 67 accessions used as female and male grandparents, and a further 13 that were used only as male grandparents (Cao et al. 2011). The number of crosses performed per accession ranged from 3 to 34, with a median of 14 F_2_ populations generated from each grandparent.

A pooled sequencing approach was employed to survey TRD in each segregating population. At least 300 individuals per F_2_ population were harvested in bulk for genotyping-by-sequencing (GBS), implemented as restriction enzyme-mediated reduced-representation sequencing (Baird et al. 2008; Monson-Miller et al. 2012). Accurate allele frequency estimate in bulks requires high sequencing coverage at each segregating site. The selected restriction enzyme, *KpnI*, cuts infrequently in the *A. thaliana* genome, allowing high coverage to be achieved for a portion of the genome, about 1%, with moderate sequencing effort. We attained an average of 78× coverage per F_2_ population (Fig. S1A), and an average of 2509 sites were segregating in any given population (Fig. S1B).

Regions displaying significant segregation distortion, as indicated by deviation from the expected 1:1 ratio of grandparental alleles, were identified by modeling the allele frequency in 5 Mb sliding windows, with 0.5 Mb steps. Non-random deviations in allele frequency estimates from pooled sequencing data can result from processes other than TRD. For example, genotype-dependent variation in seedling growth rates could result in pooled allele frequency estimates that do not reflect the genetic composition of individuals, while genotyping biases could also result from a reference-based alignment approach, where non-reference alleles might be undercalled.

To validate that our pooled sequencing approach can reliably detect TRD, we genotyped an F_2_ population (Löv-5 × Sha), where TRD had been previously reported (Salomé et al. 2012). Based on individual genotypes, TRD was observed at two genetically independent regions in this cross (Salomé et al. 2012). The Sha allele was favored on the top arm of chromosome 1, while the Löv-5 allele was preferentially inherited on the bottom arm of the same chromosome (Salomé et al. 2012). Significant TRD of both regions on chromosome 1 was replicated in our pooled sequencing data (Fig. S4). Based on modeled allele frequencies in this population, the Sha allele reached a maximum frequency of 68.6% on the top of chromosome 1. This is similar to the frequency of the Sha allele at the maximally distorted marker (70.4%) in the original study (Salomé et al. 2012). Similarly, the Löv-5 allele at the second locus reached a mean frequency of 73.8% in the pooled sequency data (compared to 73.6% in the individual genotype data) (Salomé et al. 2012). For both regions, the peak of distortion in the pooled sequencing data was within 1 Mb of the maximally distorted marker in the original study (Salomé et al. 2012).

After verifying that TRD in the Löv-5 × Sha cross was reliably detected using our pooled sequencing approach, we applied our methodology to the 492 populations passing quality control measures. In total, 62 populations (12.6%) exhibited regions of significant TRD after FDR correction for the number of tested windows (*n* = 240, *p* < 0.05) (Fig. S5). This is a rather conservative estimate of the incidence of segregation distortion in our crosses, because the ability to detect significant distortion is highly dependent on the size of the confidence interval estimates (i.e., the coverage of each population).

**Fig. 1:**
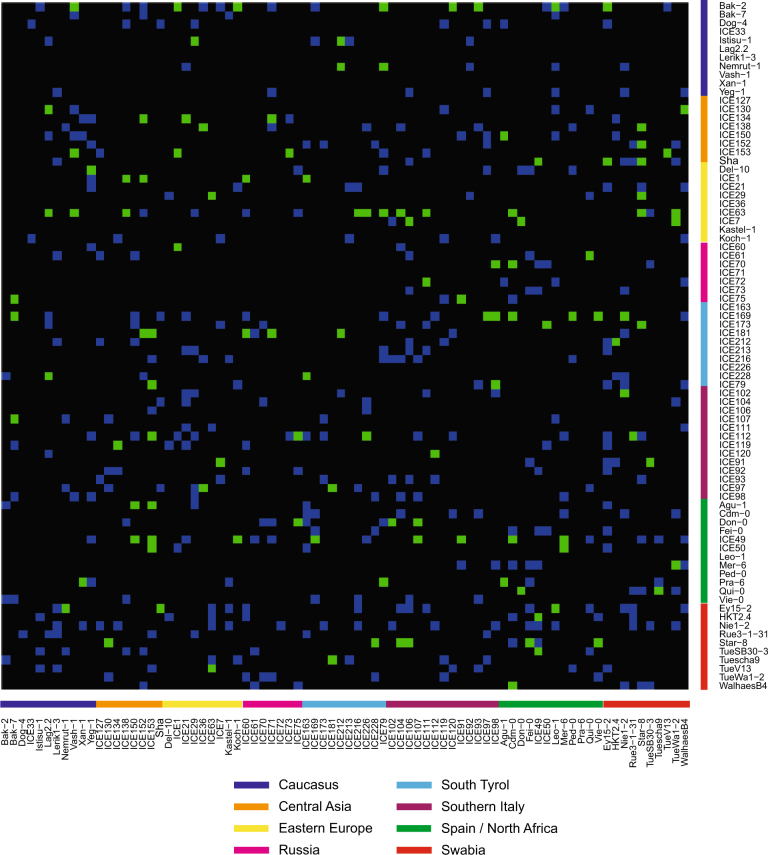
*Z*-score estimated segregation distortion is evident in a wide range of crosses. Genotypic combinations surveyed in this F_2_ screen are shown in blue, and populations with significant segregation distortion based on *Z*-score metrics in green. Grandparental accessions are ordered by the geographic location of their collection (Cao et al. 2011). Female grandparents are located on the *y*-axis and male grandparents on the *x*-axis. Intercrosses between grandparents that were not attempted are in black

To generate a less conservative estimate of the number of distorted regions, we also used a *Z*-score outlier approach. Any region with allele frequencies greater than 2.5 standard deviations from the combined population mean was considered to be distorted. This less conservative approach identified 122 (24.8%) of the 492 populations with at least a single distorted region (Fig. 1). All regions identified via the FDR method were also detected using the *Z*-score outlier approach.

**Fig. 2:**
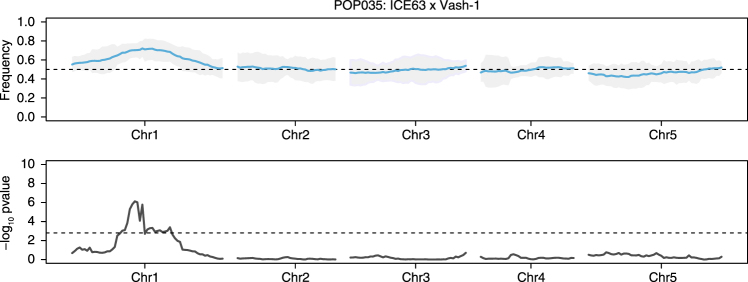
A representative F_2_ population, POP035 (ICE63 × Vash-1), with significant segregation distortion. Distortion in this population was detected with both thresholds (FDR and *Z*-score outlier). **a** The beta-binomial modeled allele frequency (blue) across each chromosome is plotted in the upper panel. 95% confidence intervals are indicated by the shaded grey area and the expected frequency of 0.5 is marked by the dashed black line. **b** The –log_10_ of the *p*-value derived from the non-parametric statistical test. The dashed black line in this panel represents the FDR corrected (*n* = 240) significance threshold (*p* < 0.05)

An example of a chromosome with a distorted region that was identified using both methods is shown in Fig. 2. Although we did not screen the complete diallel of possible F_2_ combinations, we did survey populations that sampled a large fraction of the genetic space covered by the 80 founders (Fig. 1, Fig. S5). All together, we found that TRD occurs commonly in controlled crosses between diverse *A. thaliana* accessions with evidence of significant TRD in up to 24% of surveyed F_2_ populations.

### The dynamics of segregation distortion in *A. thaliana*

**Fig. 3:**
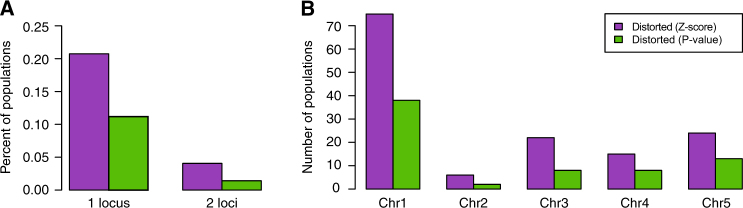
Genomic properties of distorted loci. **a** The fraction of surveyed F_2_ populations that exhibited segregation distortion at either one or two genomic loci. **b** The number of populations containing distorted loci that reside on each of the five *A. thaliana* chromosomes

Regardless of identification method—FDR or *Z*-score outlier—the majority of populations exhibited distortion at only a single locus (Fig. 3a). We also found that distortion occurs on all five chromosomes, although distorted regions are most frequently located on chromosome 1 (Fig. 3b). If TRD events were randomly distributed, we would expect to find approximately one event every 0.6–1.2 Mb (depending on the identification method). After accounting for chromosome size, there was a two-fold enrichment of TRD loci on chromosome 1 relative to the other chromosomes.

**Fig. 4:**
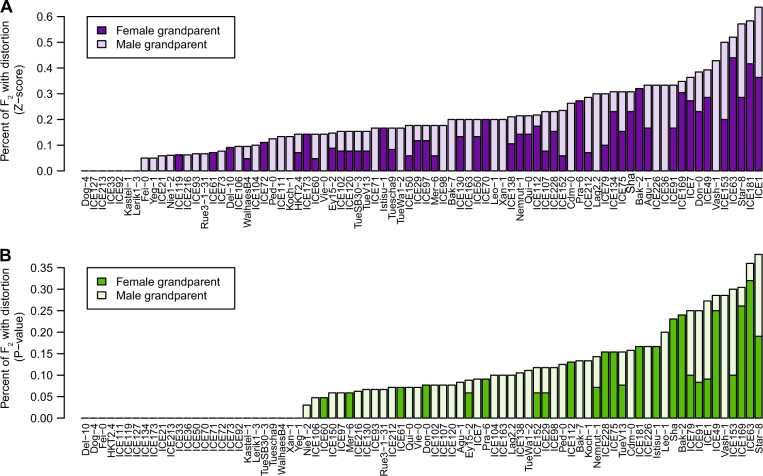
Many grandparental accessions contributed biased alleles. Each grandparent contributed its genetic material to a median of 14 distinct F_2_ populations. Plotted is the fraction of F_2_ populations with one shared grandparent that are significantly distorted as measured either by (**a**) 2.5× Z-score deviation, or (**b**) FDR corrected deviation from beta-binomial modeled allele frequencies

The alleles in distorted regions that are favored to be inherited are derived from many grandparental accessions. Of the 80 accessions used as founders, over 50 gave rise to F_2_ populations exhibiting significant segregation distortion. Some grandparents were especially notable, such as Star-8. Regions with alleles contributed by Star-8 were distorted in 60% of F_2_ populations (40% for the FDR threshold) (Fig. 4a, b).

### Refining candidate intervals surrounding distorted loci

To facilitate the genetic characterization TRD, we sought to define the minimal size of distorted genomic intervals. Genotyping F_2_ individuals in bulk enabled screening of a large number of test populations, but without genotype information from individual segregants to estimate recombination breakpoints, most candidate regions are not much smaller than entire chromosome arms.

Since we did not know a priori which populations would be the most informative to study in detail, we designed two strategies to narrow the candidate regions to facilitate subsequent fine-mapping. First, we increased the density of informative markers about 200-fold by whole-genome resequencing of six populations with severe segregation distortion. We also increased the number of recombination events in these populations by analysis of 1500 F_2_ individuals from each of the six populations. We sequenced these bulks to ∼40× coverage.

**Fig. 5:**
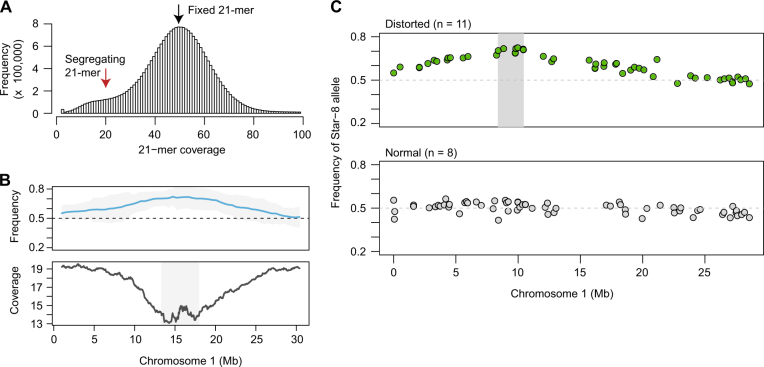
Mapping intervals refined using k-mer coverage and bulked segregant analysis. **a** The coverage of unique 21 nt k-mers is plotted for POP035 (ICE63 × Vash-1) after whole-genome resequencing. The first peak in coverage represents 21-mers found in only one of the two grandparents (red arrow), while the second, larger peak represents those sequences found in both (black arrow). **b** The upper panel displays the beta-binomial modeled allele frequency estimates (blue) and their 95% confidence intervals (grey) for POP035 as described in the legend for Fig. 2. In the lower panel, the coverage of 21-mers unique to only one of the two grandparents (coverage < 25×) is plotted in 1 Mb sliding windows (50 kb steps). Coverage decreases in the candidate regions. Intervals (grey box) are defined by merging windows with values within 1× coverage of the minimal window in each population. **c** Bulked segregant analysis was performed for Star-8, an accession that repeatedly contributed distorted loci. Sequencing reads were combined for populations exhibiting distortion when crossed with Star-8, and for populations not exhibiting distortion when crossed to Star-8 (normal pool). A candidate interval (grey box) was obtained by merging all segregating positions within 5% of the maximal allele frequency

Lower coverage at individual markers is accompanied by increased stochasticity in allele frequency estimates. We therefore took advantage of local linkage disequilibrium to diminish that noise. Short stretches of unique 21 nucleotide (nt) sequences (known as k-mers or 21-mers) were identified in the raw sequencing reads of each F_2_ population (Fig. 5a, Fig. S3). To narrow down candidate intervals, we extracted 21-mers that were predicted to be present in only one of the two grandparents. Regions of the genome that are distorted should display a decrease in coverage of such grandparent-specific 21-mers near the causal locus. Using this strategy, we were able to narrow the intervals surrounding four of the six candidate loci to less than 5 Mb, and in one case to 1.5 Mb (Table S3, Fig. 5b, Fig. S2).

In a complementary approach, we sought to refine candidate regions by obtaining a more precise estimate of local allele frequency. To this end, we greatly increased sequencing coverage by combining information from cases with shared grandparents and the same distorted regions. As mentioned earlier, some grandparental accessions contributed alleles that were favored in multiple F_2_ populations. Star-8, ICE63, and ICE49 contributed alleles that were favored in at least 40% of crosses of these to other accessions (based on the *Z*-score outlier method), with the same regions being favored in all distorted populations sharing a particular grandparent. Using a bulked segregant analysis approach (Michelmore et al. 1991), we generated two pools of reads for each grandparent. One comprised the sequencing reads from all distorted populations and the other contained the sequencing reads from all non-distorted populations.

A median coverage of at least 806× was achieved at each segregating site, vastly improving the accuracy of our estimates. For one grandparent, Star-8, we narrowed the interval to 2.0 Mb, in the middle of the top arm of chromosome 1, where recombination is high (Table S3, Fig. 5c). This strategy was less successful for the other two grandparents, ICE63 and ICE49, likely because of the distortion being less strong in these cases, as well as the location of the distorted regions near the centromere or on the distal chromosome arm, both parts of the chromosome where recombination is reduced (Table S3, Fig S6).

## Discussion

Despite the ubiquity of biased transmission of alleles in natural populations, there are few systematic studies that capture the incidence of TRD across an entire species (Corbett-Detig et al. 2013; McMullen et al. 2009; Salomé et al. 2012). Exploiting advances in sequencing and genotyping technology, we have been able to characterize segregation distortion in hundreds of intraspecific crosses. The identification of distorted regions greatly depends on sequencing coverage; in our system, a 10% deviation in absolute allele frequency becomes significant with ∼100× sequence coverage, and more subtly distorted regions could be detected with even higher coverage. Similar pooled genotyping approaches have been used to identify distorted loci in other systems (Belanger et al. 2016a, 2016b; Cui et al. 2015; Wei et al. 2017), illustrating the general power of this approach (reviewed in Schlötterer et al. 2014).

Compared to individual genotyping, one caveat of a Pool-seq approach to identify TRD is that allele frequency estimates from pooled genotyping data can be more susceptible to experimental noise. For instance, segregating variation for seedling size or germination rates can bias allele frequencies. Alignment of pooled reads to a single reference genome may also influence allele frequency estimates if one grandparental accession aligns more efficiently than the other. To estimate the extent of non-TRD influences on allele frequency estimates, we genotyped a segregating population (Löv-5 × Sha), where TRD had been previously identified via individual genotyping (Salomé et al. 2012). With 72× pooled sequencing data from the same population, we were able to confirm both TRD loci. Importantly, the predicted mean allele frequency from the pooled sequencing data was within 3% of the allele frequency estimated from individual genotype data (Salomé et al. 2012). The locations of the peaks were also coincident across data sets (within 1 Mb). In this case, our pooled genotype approach was able to accurately recapitulate the location and degree of TRD at two genomic regions suggesting that the influence of additional biases are marginal.

By surveying a broad collection of germplasm for statistical departures from Mendelian inheritance, we could confirm that allelic distortion is a common feature of F_2_ populations. Not only do distorted loci segregate in up to a quarter of all F_2_ populations, but TRD is also observed in multiple genomic regions, with the degree of distortion varying both by population and by locus, and TRD loci are contributed by over half of the 80 grandparental accessions, further emphasizing the generality of this phenomenon.

The scale of our dataset is unprecedented and this magnitude could only be achieved with the reduced cost of genotyping populations in pools. While we can confidently confirm that TRD is a common feature of segregating *A. thaliana* populations, the pooled sequencing approach comes with a few caveats. First, the detection of TRD is highly coverage dependent. While it is unlikely that strong cases of TRD were overlooked, we are unable to detect more subtle deviations in allele frequency (<10%), which could have been detected via individual genotyping (Salomé et al. 2012). In the absence of a complete account of TRD in these populations, we cannot determine if grandparents are contributing a TRD allele that is rare (i.e. distorted in only a single F_2_) or whether that allele is more common. We did identify TRD alleles that are repeatedly distorted across many populations at extreme frequencies. For example, the Star-8 region on chromosome 1 is significantly favored in ~50% of crosses, with this region being inherited by up to 70% or even 80% of the progeny. Determining the population frequency of TRD alleles is a first step to understanding the many facets of TRD, and our large-scale survey lays the groundwork for further studies by identifying crosses for more detailed follow-up experiments.

A second caveat of Pool-seq strategies is that specific location of recombination events cannot be monitored, making the resolution of allele frequency peaks a challenge. Although we were able to narrow candidate intervals to less than 8 Mb for seven specific F_2_ populations, our resolution for the remaining populations remains at the level of chromosome arms. This resolution must be improved with individual genotype data before basic questions about the evolution of TRD can be addressed. Improved mapping resolution would help to determine (1) the age of alleles (i.e. whether they are ancient alleles or have recently arisen), (2) the geographic distribution of alleles (i.e. whether TRD loci restricted to certain geographic regions), and (3) the selective forces and underlying biological process shaping TRD in this species. There is still much to be learned about the biological processes and evolutionary forces leading to uneven segregation; this large-scale survey provides a foundation to advance work on these questions.

To conclude, by surveying a large number of F_2_ populations descending from 80 genetically diverse grandparents, we were able to identify over one hundred genomic regions in *A. thaliana* that significantly deviate from the expectations of Mendelian segregation. Considering that our statistical power would not have allowed us to discover complete absence of genotypes resulting from higher-order epistatic interactions or subtle cases of single-locus TRD, it is likely that the regions we identified are only the tip of the iceberg. Notably, the majority of accessions tested contributed such distorted alleles, emphasizing the ubiquity of alleles that are unevenly transmitted. Together, these findings confirm that TRD segregating within species are more common than previously thought.

### Data archiving

Sequence data have been deposited at the European Nucleotide Archive (https://www.ebi.ac.uk/ena) under accession PRJEB27214. Genotype data have been submitted to Dryad (https://datadryad.org/): doi:10.5061/dryad.2118mj5.

## Electronic supplementary material

Figure S1

Figure S2

Figure S3

Figure S4

Figure S5

Figure S6

Table S1

Table S2

Table S3

Supplemental material - information
