# Supplementary figures and images for: Transmission ratio distortion is frequent in Arabidopsis thaliana controlled crosses
Source: Heredity (Edinb). 2018 Jun 28;122(3):294–304. doi: 10.1038/s41437-018-0107-9 (PMC6169738; doi:10.1038/s41437-018-0107-9)

**Figure S1**

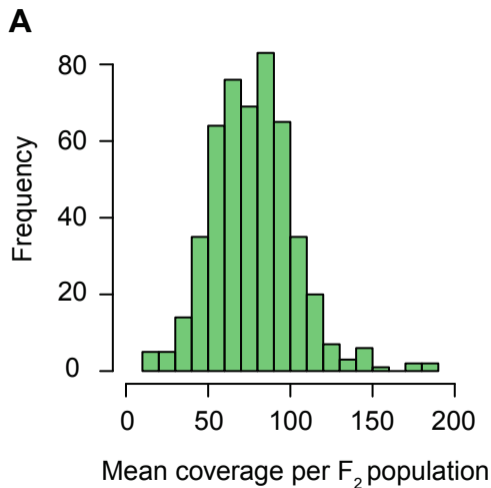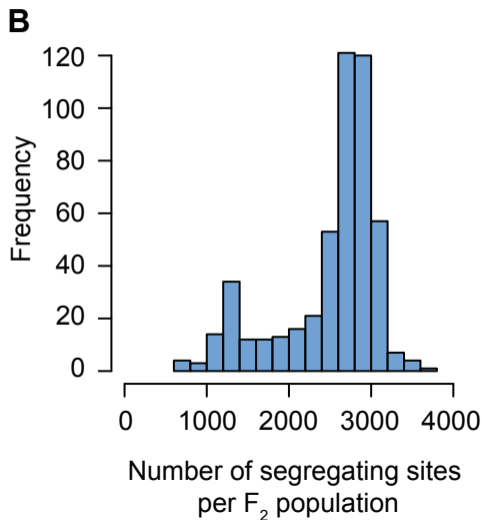

Supplement: Supplementary file 1 — Figure S1 [file 41437_2018_107_MOESM1_ESM.pdf]

**Figure S2**

POP007: ICE49 x ICE153

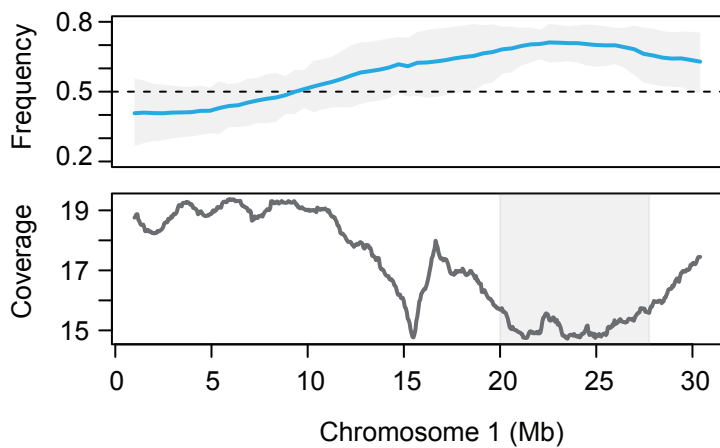

POP063: ICE169 x Bak-7

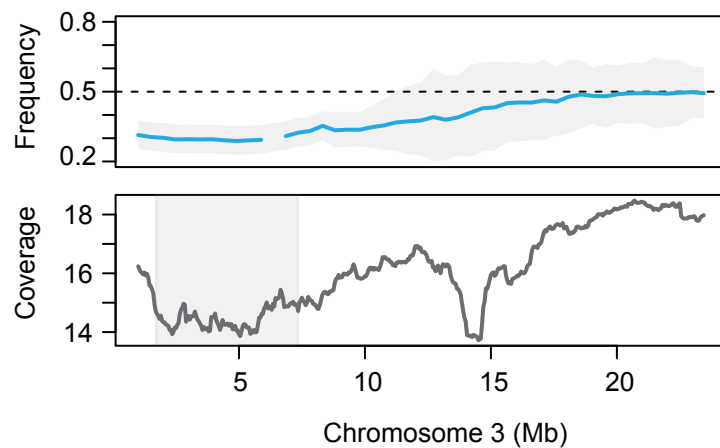

POP026: ICE63 x ICE216

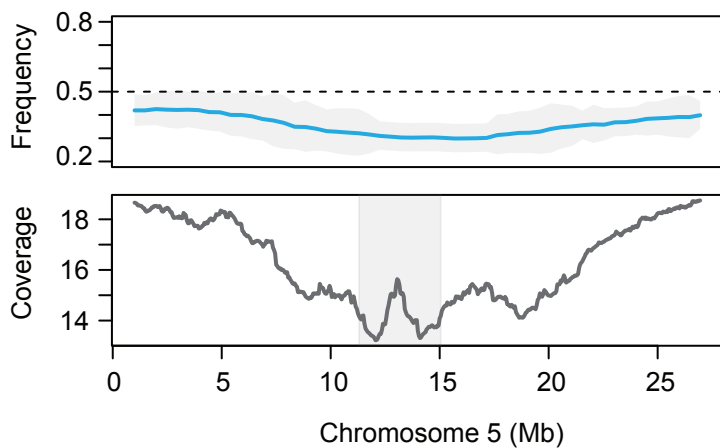

POP064: ICE169 x Cdm-0

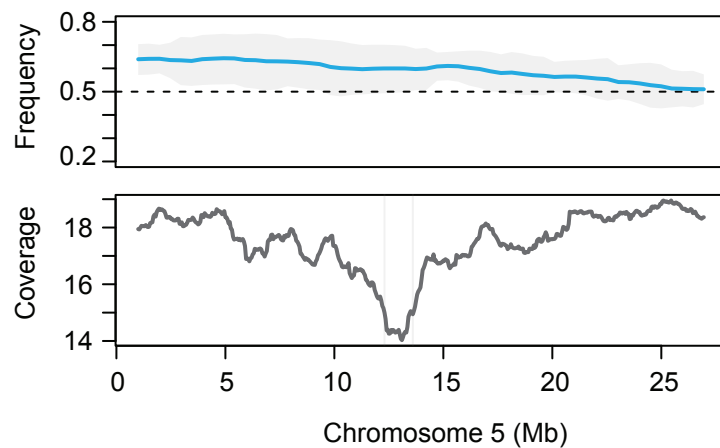

POP035: ICE63 x Vash-1

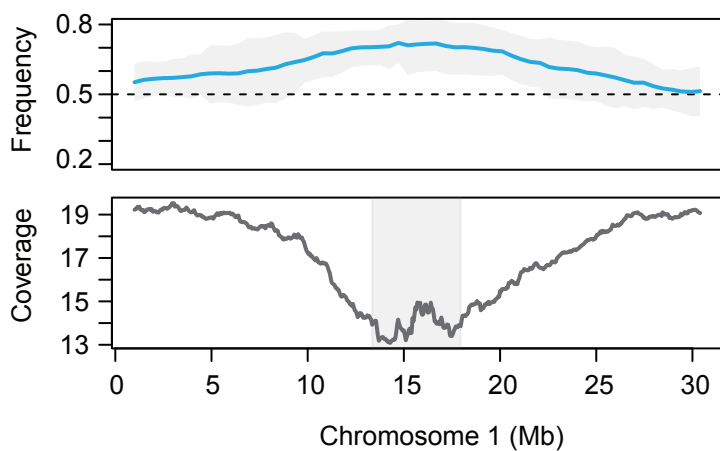

POP100: Ey15.2 x Leo-1

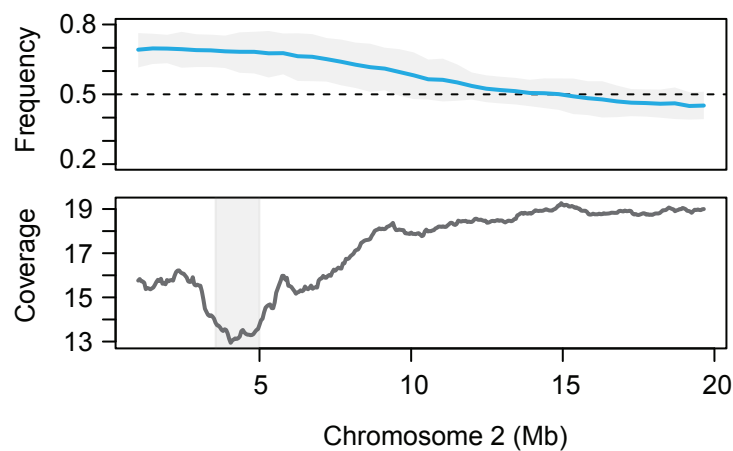

Supplement: Supplementary file 2 — Figure S2 [file 41437_2018_107_MOESM2_ESM.pdf]

**Figure S3**

POP007

POP026

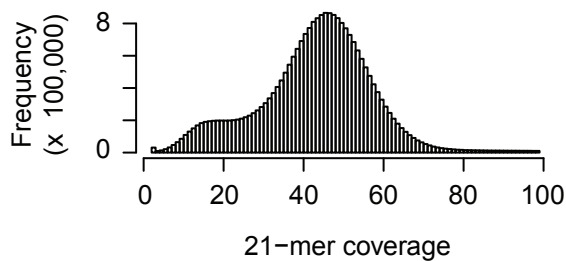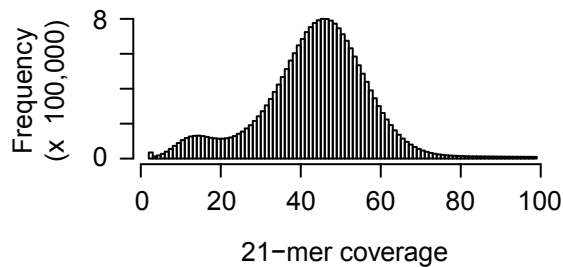

POP035

POP063

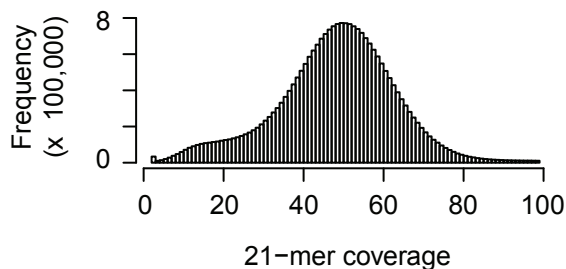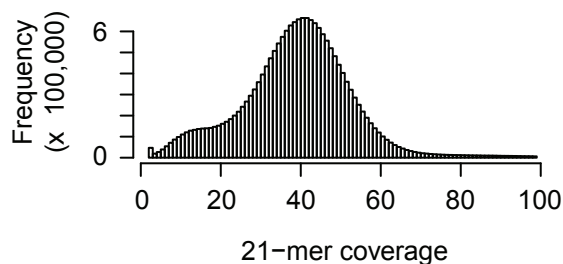

POP064

POP100

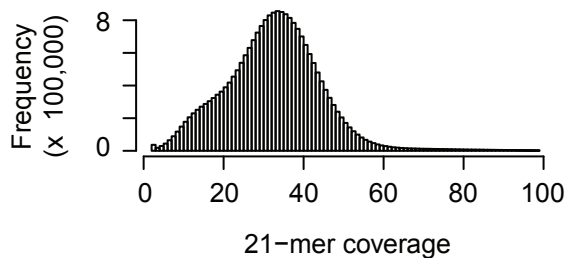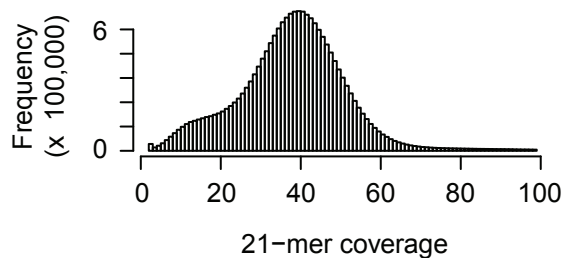

Supplement: Supplementary file 3 — Figure S3 [file 41437_2018_107_MOESM3_ESM.pdf]

**Figure S4**

Löv-5 x Sha

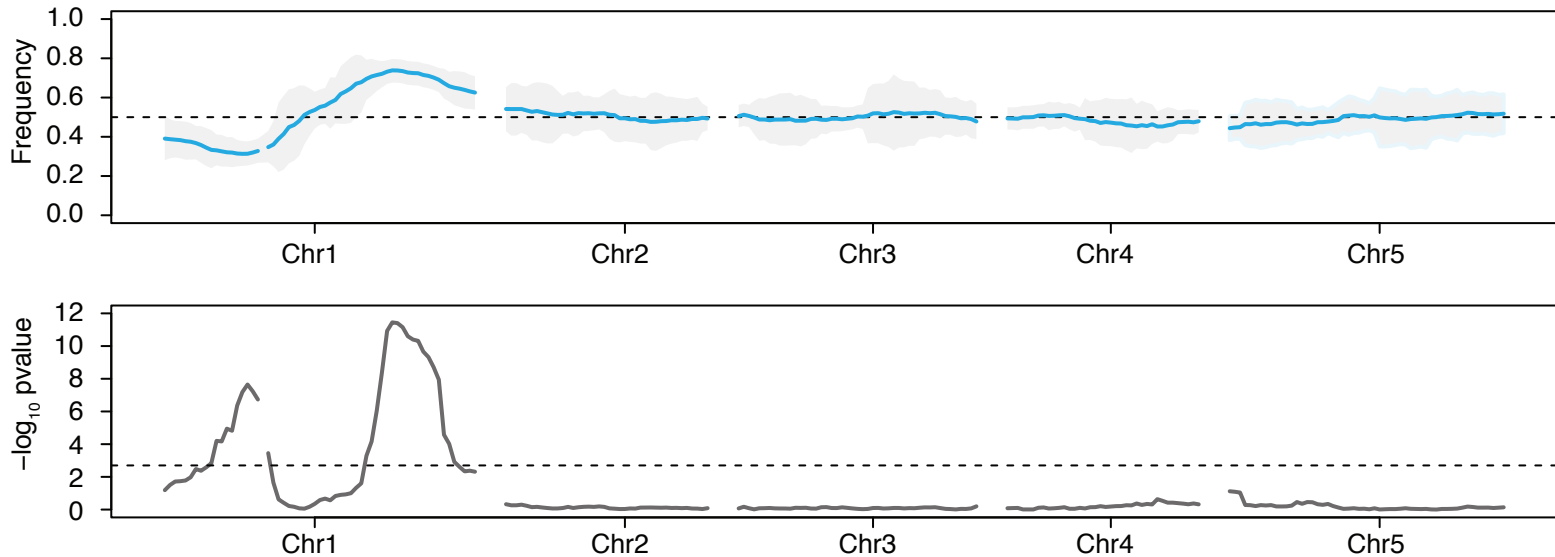

Supplement: Supplementary file 4 — Figure S4 [file 41437_2018_107_MOESM4_ESM.pdf]

Figure S5

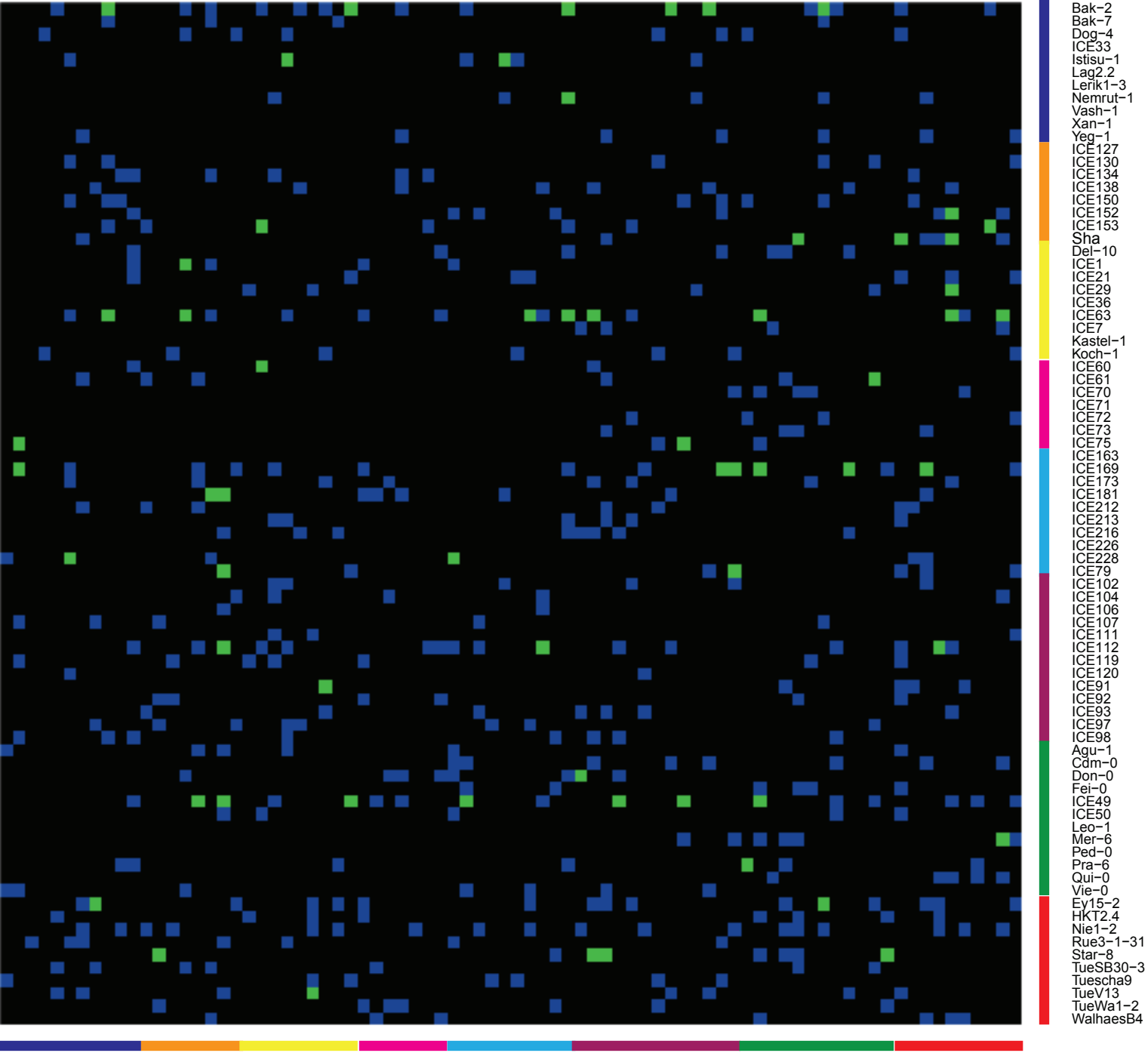

Supplement: Supplementary file 5 — Figure S5 [file 41437_2018_107_MOESM5_ESM.pdf]

**Figure S6**

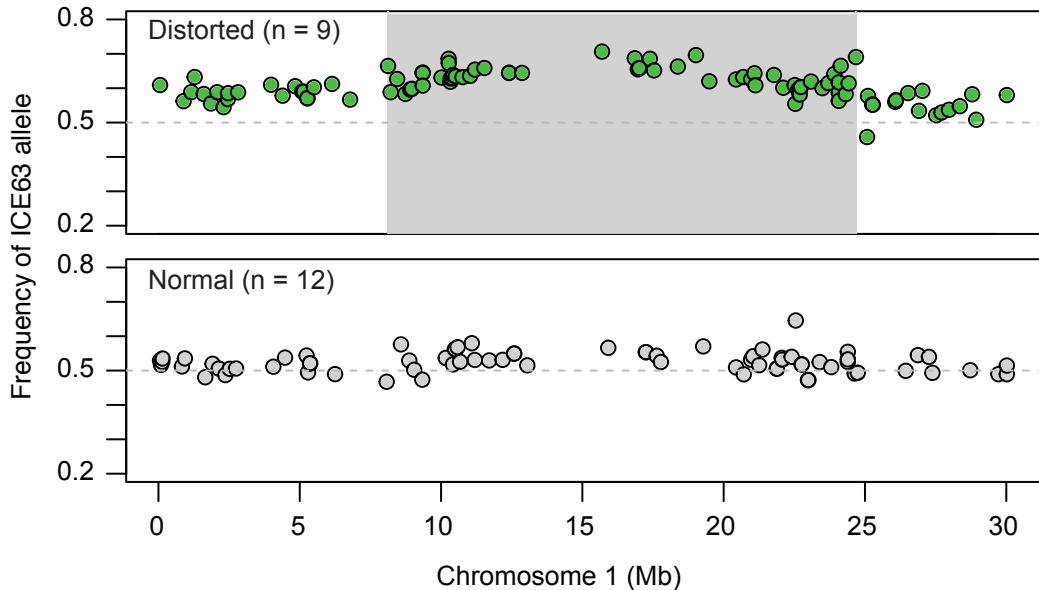

Supplement: Supplementary file 6 — Figure S6 [file 41437_2018_107_MOESM6_ESM.pdf]
