# Supplementary material for: Transmission ratio distortion is frequent in Arabidopsis thaliana controlled crosses
Source: Heredity (Edinb). 2018 Jun 28;122(3):294–304. doi: 10.1038/s41437-018-0107-9 (PMC6169738; doi:10.1038/s41437-018-0107-9)
Supplement: Supplementary file 10 — Supplemental material - information [file 41437_2018_107_MOESM10_ESM.docx]

# Supplemental information

# Supplemental tables

**Table S1. Germplasm information for surveyed F_2_ populations.** All crosses are listed, with those passing quality control (QC) indicated with a “1”. Similarly, “1” and “0” indicates whether distortion was detected using FDR significance testing of beta-binomial modeling of allele frequencies or Z-score deviation.

**Table S2. Germplasm.** Correspondence between grandparental accession names and CS accession IDs.

**Table S3. Candidate intervals for distorted loci.** ND, not determined.

# Supplemental figure legends

**Figure S1. Reduced-representation sequencing reliably enriches for 1% of the *A. thaliana* genome.** (A) Mean sequencing coverage at sites segregating in each F_2_ population. (B) Number of sites segregating in each F_2_ population. The mean observed number of segregating sites (2,500) is comparable to the expected number of segregating sites derived from previously published resequencing data (Cao *et al*, 2011).

**Figure S2. 21-mer coverage from whole-genome resequencing can be used to refine mapping intervals.** For each population, the upper panel displays the beta-binomial modeled allele frequency estimates (blue) and their 95% confidence intervals (grey) as described in the legend for Figure 2. In the lower panel, the coverage of 21-mers unique to only one of the two grandparents (coverage < 25x) is plotted in 1 Mb sliding windows (50 kb steps). Coverage decreases in the candidate regions. Intervals (grey box) are defined by merging windows with values within 1x coverage of the minimal window in each population. No candidate region was defined for POP064 as coverage decrease coincides with the centromere, not the distorted region.

**Figure S3. Distribution of unique 21-mers in whole-genome resequencing data.** The coverage of unique 21 nt k-mers is plotted for each of the six populations that underwent whole-genome resequencing. The first peak in coverage represents 21-mers found in only one of the two grandparents, while the second, more prominent peak represents those found in both.

**Figure S4. Significant segregating distortion in a control population (Löv-5 x Sha).** Distortion in this population was previously detected based on individual genotyping (Salomé *et al*, 2012). (A) The beta-binomial modeled allele frequency (blue) across each chromosome is plotted in the upper panel. 95% confidence intervals are indicated by the shaded grey area and the expected frequency of 0.5 is marked by the dashed black line. (B) The –log_10_ of the p-value derived from the non-parametric statistical test. The dashed black line in this panel represents the FDR corrected (n = 240) significance threshold (p < 0.05).

**Figure S5. Statistically significant segregation distortion is evident in a wide range of crosses.** Genotypic combinations surveyed in this F_2_ screen are shown in blue, and populations with significant segregation distortion based on non-parametric statistical tests of beta-binomial modeled allele frequencies in green. Grandparental accessions are ordered by the geographic region of their collection (Cao *et al*, 2011). Female grandparents are located on the y-axis and male grandparents on the x-axis. Intercrosses between grandparents that were not attempted are in black.

**Figure S6. Increasing the number of analyzed segregants can be used to refine mapping intervals.** Bulked segregant analysis was performed for grandparental accessions that repeatedly contributed distorted loci (Star-8 [Figure 6C], ICE63 [shown here], and ICE49). Sequencing reads were combined for populations exhibiting distortion or not exhibiting distortion when crossed to the focal grandparent. An average of over 800x coverage was achieved at sites segregating between the focal accessions and all other members in the bulk. A candidate interval (grey box) was obtained by merging all segregating positions within 5% of the maximal allele frequency. Data for ICE49 not shown, as there were too few segregating sites.
